# Stroke Telerehabilitation in Calabria: A Health Technology Assessment

**DOI:** 10.3389/fneur.2021.777608

**Published:** 2022-01-07

**Authors:** Marianna Contrada, Francesco Arcuri, Paolo Tonin, Loris Pignolo, Tiziana Mazza, Giuseppe Nudo, Maria Luigina Pignataro, Maria Quintieri, Antonella Iozzi, Antonio Cerasa

**Affiliations:** ^1^S. Anna Institute, Crotone, Italy; ^2^Institute for Biomedical Research and Innovation, National Research Council (IRIB-CNR), Messina, Italy; ^3^Preclinical and Translational Pharmacology, Department of Pharmacy, Health Science and Nutrition, University of Calabria, Cosenza, Italy

**Keywords:** telerehabilitation, stroke, motor and functional recovery, virtual reality rehabilitation system, rehabilitation

## Abstract

**Introduction:** Telerehabilitation (TR) is defined as a model of home service for motor and cognitive rehabilitation, ensuring continuity of care over time. TR can replace the traditional face-to-face approach as an alternative method of delivering conventional rehabilitation and applies to situations where the patient is unable to reach rehabilitation facilities or for low-income countries where outcomes are particularly poor. For this reason, in this study, we sought to demonstrate the feasibility and utility of a well-known TR intervention on post-stroke patients living in one of the poorest indebted regions of Italy, where the delivery of rehabilitation services is inconsistent and not uniform.

**Materials and Methods:** Nineteen patients (13 male/6 female; mean age: 61.1 ± 8.3 years) with a diagnosis of first-ever ischemic (*n* = 14) or hemorrhagic stroke (*n* = 5), who had been admitted to the intensive rehabilitation unit (IRU) of the Institute S. Anna (Crotone, Italy), were consecutively enrolled to participate in this study. After the discharge, they continued the motor treatment remotely by means of a home-rehabilitation system. The entire TR intervention was performed (online and offline) using the Virtual Reality Rehabilitation System (VRRS) (Khymeia, Italy). All patients received intensive TR five times a week for 12 consecutive weeks (60 sessions, each session lasting about 1h).

**Results:** We found a significant motor recovery after TR protocol as measured by the Barthel Index (BI); Fugl-Meyer motor score (FM) and Motricity Index (MI) of the hemiplegic upper limbs.

**Conclusions:** This was the first demonstration that a well-defined virtual reality TR tool promotes motor and functional recovery in post-stroke patients living in a low-income Italian region, such as Calabria, characterized by a paucity of specialist rehabilitation services.

## Introduction

Stroke is one of the most important causes of death (1) and disability worldwide (2). The decline of mortality (3) contributed to long-term outcomes, such as sensory, motor, cognitive, and visual impairments, representing great challenges to be addressed by the survivors.

The presence of disabilities following a stroke has also an important impact on society in terms of costs for the healthcare system as well as for the quality of life of patients and their families (4). The amount of time in acute care after stroke is getting shorter and the rehabilitative phase is increasingly shifted toward an outpatient setting. Nevertheless, the successive discharge does not always coincide with an adequate functional recovery, which requires longer time and many resources (5). Moreover, since there are limited healthcare resources and infrastructures, stroke survivors often receive medical assistance according to nonclinical factors, such as geographical location and personal wealth (6–8). This is particularly evident in Italy, where the chronic public health management crisis is exacerbated in the poorest southern areas, such as Calabria, where the delivery of post-stroke rehabilitation services is not uniform, inconsistent, and deeply indebted. Furthermore, traveling to hospital services is extremely challenging for both rural and urban dwellers with disabilities, due to the mountainous terrains of the region and the limited transport facilities. All these factors contributed to health migration from the Calabria healthcare system.

For this reason, it is mandatory to identify new effective and efficient models of care to improve the increasing demand for stroke rehabilitation services, considering the complex geographical and territorial socio-economic problems in Calabria.

In recent years, telemedicine has also been used as a method of intervention and support to families of patients with stroke (9). Technologies related to remote health services also offered an alternative solution for extending neurological interventions to patients who do not live near a qualified care provider, thus, addressing inequalities in access to health (10). Telephone, internet-based video conferencing, virtual reality protocols, and sensors can be used as means of communication between patients and healthcare experts, providing an alternative and effective method of delivering conventional rehabilitation (5, 11, 12). Telerehabilitation (TR) can ensure the continuity and/or prolong the treatment that started in rehabilitation units (13) and improve, as an additional treatment, the quality and the amount of conventional therapy. Moreover, different types of rehabilitative treatments can be delivered by TR systems, like physiotherapy, speech, occupational, and cognitive therapy, besides teleconsultations (14). Finally, it has been demonstrated that the neurofunctional changes underlying recovery from stroke are similar in patients who underwent conventional rehabilitation, as well as TR (5, 15, 16). For all these reasons, TR appears as a promising and effective intervention for neurological patients, supporting the healthcare system, and going beyond territorial difficulties and isolation (4).

This study aims to evaluate, for the first time, the utility and feasibility of a well-known and validated TR tool for post-stroke patients living in a low-income Italian region such as Calabria.

## Materials and Methods

### Participants

The study was realized on post-stroke patients who required long-term motor/cognitive assistance, consecutively enrolled, from January 2020 to May 2021, at the time of their discharge from the Intensive Rehabilitation Unit (IRU) at the S. Anna Institute (Crotone, Italy). The inclusion criteria were: (1) Age > 18 years; (2) stable clinical condition; (3) absence of infections; (4) availability of receiving in-home neuro-rehabilitation service; and 5) availability of a home internet connection. The exclusion criteria were: (1) patients with a history of regular prior and/or current drug and/or alcohol abuse; (2) patients with cognitive impairment (Mini Mental State Examination (MMSE) score > 24); (3) patients with aphasia, as assessed by the Aachener Aphasie Test (AAT); (4) prior or current psychiatric diseases; and (5) presence of other severe pathologies influencing the outcome, such as cardiorespiratory instability or other medical illness potentially interfering with the treatment.

All the participants gave written informed consent. The study was approved by the Ethical Committee of the Central Area Regione Calabria of Catanzaro (prot n° 168; 20/07/2017), according to the Helsinki Declaration.

### Design and Procedure

**Figure 1:**
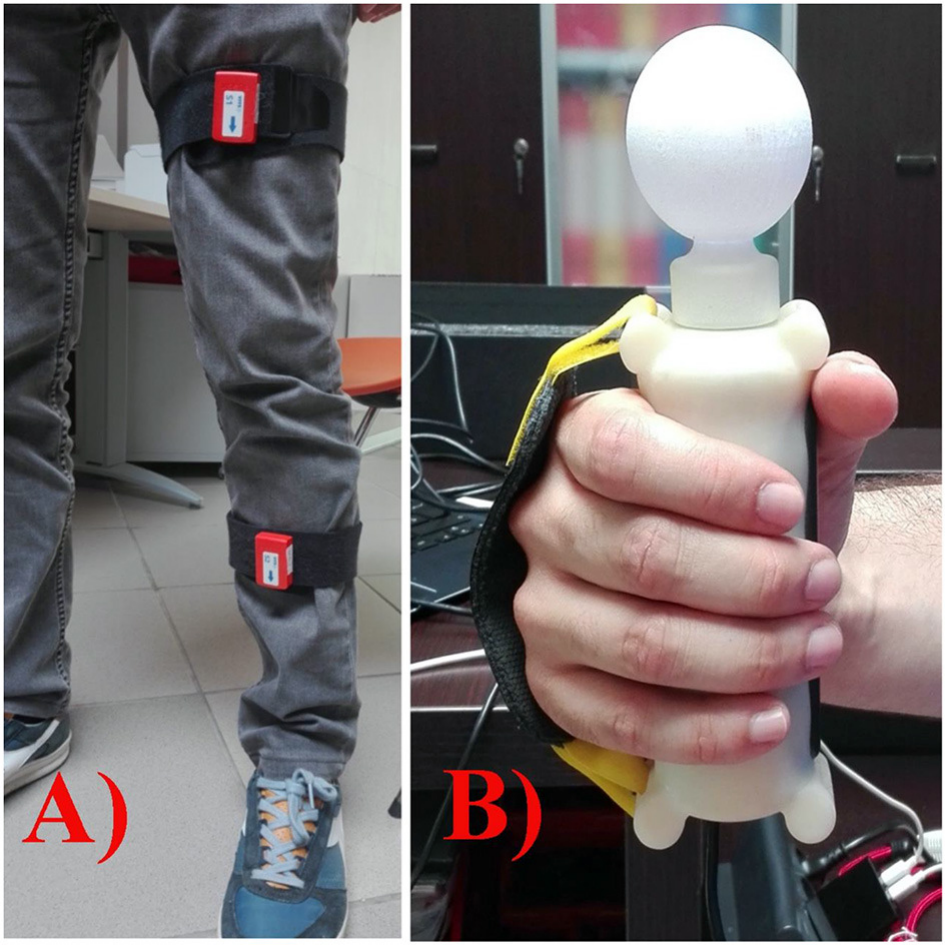
Khymu **(A)** and KWand **(B)** devices for telerehabilitation.

A within-subject design divided into four main stages was used. After the recruitment of the patients (1° phase), in the second stage, occupational and physical therapists blindly assessed the clinical scales before and after treatment. Next, the participants underwent neuro-rehabilitation training for five times a week for 1-h per session in 12 consecutive weeks. For remote rehabilitation treatment, we used the Virtual Reality Rehabilitation System (VRRS), which is recognized as one of the most advanced, comprehensive, and clinically employed virtual reality systems for rehabilitation (14, 17) and TR (18, 19). This is a technological innovation tool that allows delivering motor (13), cognitive, and speech (19, 20) neurological treatments *via* remote advanced technological devices (23). It is designed as a hub-and-spoke system and includes the telecockpit (hub) and the home tablet (spoke) (http://khymeia.com). The therapist by the VRRS Telecockpit (workstation) manages the home tablet and remotely guides the patient training. The VRRS tablet is delivered to the patient and contains the exercises to be performed by means of sensors (Khymu and K-Wand) (Figures 1A,B). For telerehabilitation protocols, the VRRS system provides a non-immersive virtual reality (VR) tool at home, which patients can navigate using a keyboard/mouse into a virtual environment displayed on a computer screen. All motor and logopedic exercises are tailored to the clinical status of stroke patients. More specifically, the motor exercises included in the software reflect those of in-patient rehabilitation programs administered during the conventional treatment. Applying the sensors (khymu) both to the trunk and the upper/lower limbs, or reaching the target holding the K-wand, it is possible to train the different body segments.

The software contains specific exercises for the trunk (flexion and extension, rotation, lateral inclination, and dorsal mobilization), for the singular right or left upper limb (shoulder adduction and abduction, shoulder flexion and extension, shoulder intra- and extrarotation, elbow flexion and extension, and forearm pronation and supination), and for singular (hip abduction, adduction, flexion and extension, knee flexion and extension, and ankle flexion and extension) or both lower limbs (squats, get up on the tips, and march on the spot). Other exercises, namely, “functional exercises” reproduced activities of daily living (ADLs) such as ironing, opening a jar, and bringing a glass to the mouth. Furthermore, the same exercises can be performed in two modalities: online (the therapist controls the tablet and interacts with the patient) or offline (the patient carries out on his/her own exercises), by using a system of non-immersive VR.

Prior to rehabilitation, the patients were trained to the employment of the VRRS Tablet. Since the pandemic restrictions, both assessment and training for the use of the VRRS tablet were remotely conducted.

Finally, Khymeia provides an efficient system that captures all necessary information for remote medical services which meets all our security and privacy requirements, i.e., patient's privacy (anonymity), data confidentiality, and data integrity.

### Clinical Evaluation

The Fugl-Meyer (FM) (21, 22) and the Motricity Index (MI) scales were used to assess the motor functions. The first one was used to assess the motor performance of the upper and lower limbs, as well as control of sitting and standing balance, while the second scale measures the strength in the upper and lower paretic extremities (23). The Barthel Index (BI), developed as a measure to assess disability in patients with neuromuscular and musculoskeletal conditions, was used to assess the independence in the ADL (23). The possible presence of depressive symptoms was investigated by means of the Post-Stroke Depression Rating Scale (PSDRS), specifically designed for patients with stroke (24).

A satisfaction questionnaire was included in the protocol and was sent by email at the end of the treatment to the patients/caregiver who enrolled in the study. The purpose of this questionnaire was to find out their opinion and the difficulties and satisfaction degree in relation to the TR service. The questionnaire was structured as a Likert scale and included 9 items: 1–5 multiple answers, scored from lowest to the highest level of satisfaction (not much – 1; enough – 2; much – 3; very much - 4); 6 and 7 closed answers (Yes or No); and 8 and 9 open answers (for more information see Supplementary Materials).

### Statistical Analysis

Data analyses were performed using SPSS version 23.0 (IBM, New York, USA). The assumptions for normality were tested for all continuous variables using the Kolmogorov–Smirnov test. Clinical variables were normally distributed. A paired-sample *t*-test (two-sided) was used to verify any statistically significant changes in motor scales before and after TR treatment. For all tests, a *p*-value < 0.05 was statistically significant.

## Results

### Patients

**Figure 2:**
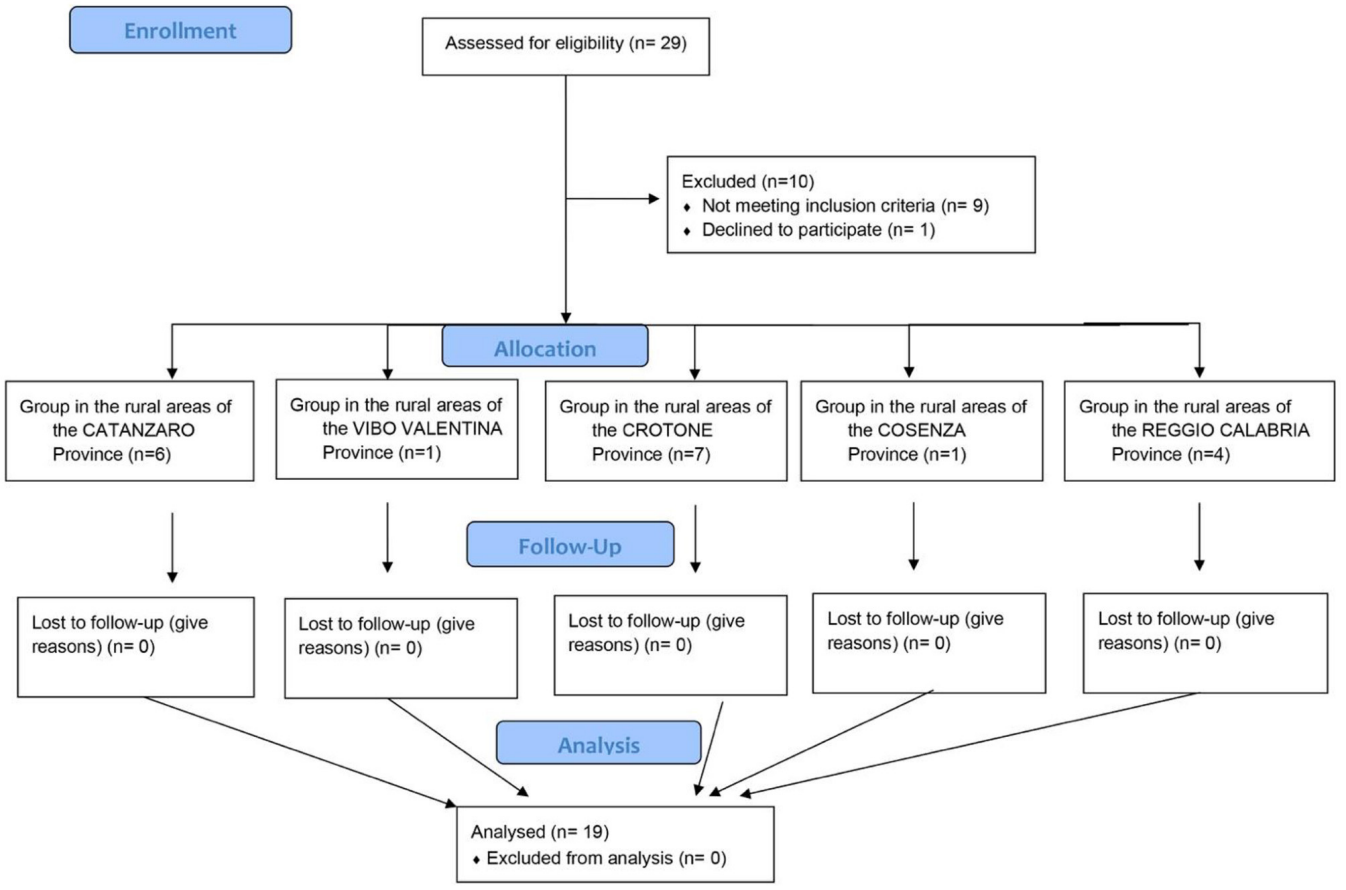
Flow diagram showing the phases of parallel trial of patients with stroke who underwent TR treatment in Calabria provinces.

**Figure 3:**
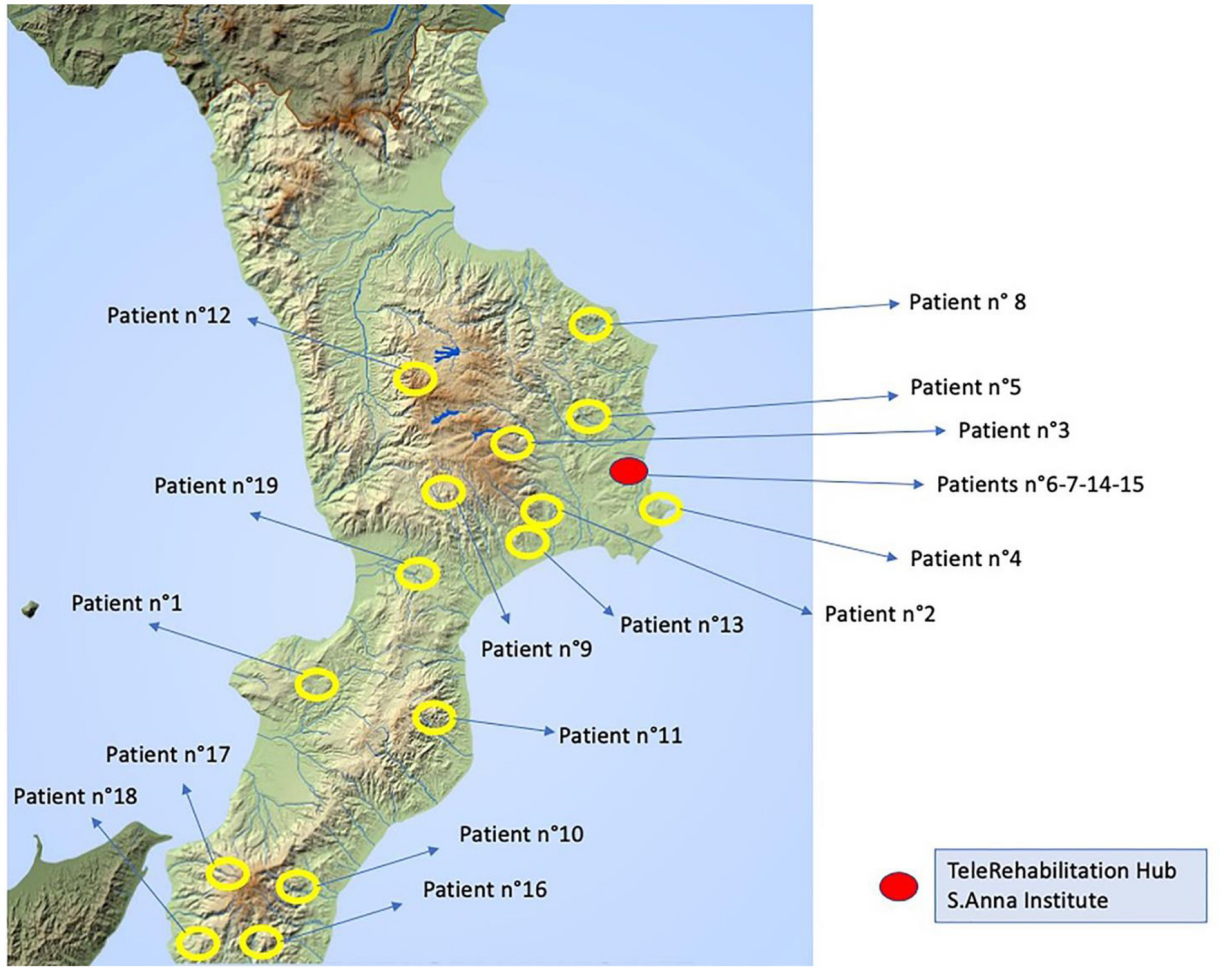
Physical map of the Calabria region showing patients' localizations during TR treatment.

Nineteen stroke patients (ischemic, *n* = 14; hemorrhagic stroke, *n* = 5) (Table 1) completed all phases with a higher level of adherence to the home tele-treatment and were included in the statistical analysis (Figure 2). The TR protocol has included patients in the entire Calabria region (Figure 3). The qualitative evaluation of personal satisfaction opinion about TR showed a very good level with the following mean percentages: (i) “Excellent” 64.6%; “Very Good” 24.8%; “Satisfactory” 16%. One hundred percent of the interviewed patients answered positively to items 6 and 7, while the problems encountered during the treatments were only related to the internet speed connection.

**Table 1:** Demographic and clinical data at admission to telerehabilitation (TR) protocol.

**Variables**	
Number	19
Sex (% male)	68%
Age (years)	61.1 ± 8.3 [44–73]
Educational Level (years)	12.4 ± 4.3 (5–18)
Time from event (days)	595 ± 688.3 [43–3,396]
Etiology	73.6% Ischemic
	26.4% Hemorrhagic
MMSE	25.86 ± 3.0 [24–29.4]
*PSDRS*	2.74 ± 2.8 [0–9]

*Data are shown as mean ± SD including minimal and maximal values. MMSE, Mini-Mental State Examination; PSDRS, Post-Stroke Depression Rating Scale*.

### Motor Outcome After TR

To longitudinally assess the motor recovery after TR protocol, a comparison within groups was performed (Table 2). We found a significant improvement in the BI, FM, and MI performance of the left hemiplegic upper limbs. A trend toward relevant improvement was also detected in the right hemiplegic upper/lower limbs, without reaching a significant threshold.

**Table 2:** Clinical improvements within post-stroke patients before (pre-) and after the (post-) TR protocol.

	**Pre-TR**	**Post-TR**		
	***(n = 19)***	***(n = 19)***	***p-value***	***t-value***
BI	72.11 ± 22.7	81.8 ± 17.6	0.002^*^	−3.74
FM	74.53 ± 22.7	79.3 ± 23.6	0.001^*^	−3.77
MI upper limbs *(paretic limb)*	68.6 ± 24.1	71.32 ± 23.6	0.02^*^	−2.73
MI upper limbs (non-*paretic limb)*	90.21 ± 20.5	91.63 ± 18.5	0.06	−1.82
MI lower limbs *(paretic limb)*	72.84 ± 20.7	72.26 ± 20.3	0.34	−0.94
MI lower limbs *(non-paretic limb)*	88.61 ± 20.2	93.05 ± 12.8	0.11	−1.7

*TR, Telerehabilitation protocol; BI, Barthel Index; FM, Fugl-Meyer; MI, Motricity Index. Data are shown as mean ± SD*.

* *Significant at p < 0.05*.

## Discussion

This study assessed the feasibility and the utility of a TR approach for providing effective intervention in post-stroke patients living in a low-income Italian region. Our findings confirmed that during a TR protocol, better motor and functional recoveries were detected after discharge from the hospital. Despite the lack of a control group that did not allow us to quantify the real magnitude of recovery, our study supports the use of this advanced technological tool to improve the access to rehabilitation services.

Telemedicine is generally underdeveloped in Italy and disparities exist among the different regions with regard to the provision of healthcare (4). The increasing commitment of resources for the national healthcare system has reached a dramatic condition, leading some regions to be placed under temporary administration. Calabria is one of the most indebted regions, as also characterized by the presence of several rural places and paucity of specialist rehabilitation services. For this reason, urgent efforts are needed to promote early and continuous interventions at distance and to reduce the long waiting lists and the costs for the healthcare system. To provide an objective quantification of the actual healthcare system status in Calabria, Guerriero et al. (25) described the reorganization problems faced by local authorities in defining the hospital network of the region of Calabria. Based on real data used for evaluating the status of public health care service network (i.e., number of hospitals, number of beds in the department, connection cost expressed in terms of travel time between home and the hospital location; and demand from center), they compared the current hospital network in Calabria with the ideal localization obtained by solving some classical facility computational location models. The results showed that the current hospital network configuration in Calabria leaves some of the demand uncovered, thus, generating inefficiencies for the healthcare system that may also directly impact stroke patients requiring access to rehabilitation services.

As recently reviewed by Maresca et al. (4), there are few studies that were carried out in Italy to evaluate the reliability and efficacy of TR protocols on stroke patients. Using the Human Empowerment Aging and Disability rehabilitation program, Isernia et al. (26) demonstrated in a heterogeneous neurological population of Northern Italy (n° 30 with Parkinson's Disease, n° 32 with Multiple Sclerosis, and n° 45 with stroke in chronic stage) that TR is effective in ameliorating autonomy in daily routines. Torrisi et al. (27) evaluated the efficacy of TR on 40 patients with post-stroke who were living in Sicily and were randomly divided into experimental and control groups. The experimental group underwent cognitive rehabilitation training using the VRRS-Evo, whereas, the control group performed a traditional rehabilitation program. After an initial training phase performed during the IRU period, the patients continued rehabilitation at home with a tablet remotely connected with clinicians. These authors found a significant increase in global cognitive functioning, attentional processes, verbal fluency, short-term memory, and mood (anxiety and depression) after TR protocol with respect to the control group. Finally, in two distinct works, Piron et al. (28, 29) demonstrated the effectiveness of TR using a virtual reality-based system delivered *via* the Internet to induce the motor recovery of the upper limbs in post-stroke patients. In particular, they found a significant increase of performance only in the FM scale with respect to the control group who underwent traditional physical therapy for the upper limbs. They claimed that TR can stimulate learning of the arm's motor skills away from the healthcare facilities, with reduced healthcare costs.

Our study is perfectly in agreement with the literature showing that a well-validated TR tool induces motor and functional recovery in chronic post-stroke patients. The telemedicine system employed in this study is the VRRS. This tool has been developed in the last few years for the TR of a wide spectrum of pathologies, demonstrating high feasibility in promoting motor or cognitive recovery in patients with multiple sclerosis (30) or Parkinson's disease (31). The strengths of VRRS are as follows: (a) the modular organization integrating telerehabilitation games and exercises in addition to teleconsulting, with remote territorial and home control (32); and (b) the possibility to employ different rehabilitation modules for training motor, cognitive and orthopedic deficits. This is achieved by the interaction between the virtual system, therapist, and patient with a series of synchronized and integrated customizable devices, adapting to the patient's disabilities and needs.

### Limitations

The small sample size and the lack of a control group are the main limitations of this study. Although we are aware of these limitations, it is important to bear in mind that the effectiveness of TR in recovering motor/functional abilities with respect to conventional treatment has previously been demonstrated in several neurological domains (4). The main target of this study was to demonstrate, for the first time, that this healthcare system might also be applied in a low-income Italian region rather than evaluating its effectiveness with respect to other approaches. Thus, our study should be considered as a pilot, with promising future applications to overcome the barriers related to access to services caused by distance or difficulty of patient's mobility in Calabria (25).

## Conclusions

A clear advantage of TR is to increase access for people living in isolated or far from rehabilitation services. This means that a TR service makes a competent rehabilitation team available for all the geographical zones, where the technology is present but the access to health services is complex or lacking (rural and remote regions), and for all people with restricted mobility (5). In this study, we demonstrated that TR interventions for functional recovery may be successfully delivered in patients with post-stroke, overcoming geographical and organizational barriers characterizing the healthcare configuration of Calabria. Our study also highlights the need for additional investments in community-based stroke rehabilitation services to make this new model of care suitable and available for a larger group of post-stroke patients (32).

## Data Availability Statement

The raw data supporting the conclusions of this article will be made available by the authors, without undue reservation.

## Ethics Statement

The study was approved by the Ethical Committee of the Central Area Regione Calabria of Catanzaro (prot n° 168; 20/07/2017) in accordance with the Helsinki Declaration. The patients/participants provided their written informed consent to participate in this study.

## Author Contributions

The statistical analysis was done by MC. The study design was done by MC, FA, PT, and AC. The manuscript drafting was done by MC, FA, and AC. Clinical data collection was made by FA, TM, GN, MLP, AI, and MQ. The literature search, data interpretation, and manuscript revision were done by FA, MC, LP, PT, and AC. All authors contributed to the article and approved the submitted version.

## Conflict of Interest

The authors declare that the research was conducted in the absence of any commercial or financial relationships that could be construed as a potential conflict of interest.

## Publisher's Note

All claims expressed in this article are solely those of the authors and do not necessarily represent those of their affiliated organizations, or those of the publisher, the editors and the reviewers. Any product that may be evaluated in this article, or claim that may be made by its manufacturer, is not guaranteed or endorsed by the publisher.
